# Retraction Note: Silencing of long non-coding RNA TUC338 inhibits the malignant phenotype of nasopharyngeal cancer cells via modulating the miR-1226-3p/FGF2 axis

**DOI:** 10.1007/s12672-024-01466-3

**Published:** 2024-10-23

**Authors:** Jian Wang, Liping Li, Xue Jiang, Bin Wang, Xiaodong Hu, Weiwei Liu, Ying Zhang

**Affiliations:** 1 Department of Otolaryngology, Cangzhou Central Hospital, No. 16 Xinhua West Road, Cangzhou, 061000 Hebei China; 2 Infection Department, Cangzhou Central Hospital, No. 16 Xinhua West Road, Cangzhou, 061000 Hebei China

**Retraction note: Discover Oncology (2022) 13:102** 10.1007/s12672-022-00544-8

The authors have retracted this article. After publication they found that some participants had already received treatment in other hospitals before the collection of their tissue for analysis in this study and so did not meet the inclusion criteria. The results and conclusions presented may therefore not be reliable. All authors agree with this retraction.

